# Retrospective Analysis of Drinking Water Microcosm Microbiomes Reveals an Apparent Antagonistic Relationship between and 

**DOI:** 10.1021/acs.estlett.5c00590

**Published:** 2025-07-17

**Authors:** Fernando A. Roman, Thomas Byrne, Rebekah L. Martin, Didier Mena-Aguilar, Rania E. Smeltz, Rachel Finkelstein, Amy Pruden, Marc A. Edwards

**Affiliations:** † Department of Civil and Environmental Engineering, 551521 Virginia Tech, Blacksburg, Virginia 24061, United States; ‡ Department of Genetics, Bioinformatics, and Computational Biology, 1757 Virginia Tech, Blacksburg, Virginia 24061, United States; § Department of Civil and Environmental Engineering, Virginia Military Institute, Lexington, Virginia 24450, United States; ∥ Department of Biochemistry, 14719 University of Nebraska-Lincoln, N106, The Beadle Center, Lincoln, Nebraska 68588-0664, United States; ⊥ Department of Microbiology, University of Alabama at Birmingham, Birmingham, Alabama 35294-2170, United States; # AECOM, 3101 Wilson Boulevard, Arlington, Virginia 22201, United States

**Keywords:** probiotics, premise plumbing, drinking water, *Neochlamydia*, *Legionella*, microbial ecology

## Abstract

(*Lp*) can sometimes establish in drinking water microbial communities and infect individuals inhaling contaminated aerosols. The premise plumbing portion of the drinking water distribution system is often especially vulnerable to *Lp* growth. Innovative approaches to intentionally manipulate the microbial ecology to control *Lp* have been proposed but remain elusive. Here, we retrospectively analyzed 16S rRNA gene amplicon sequences and droplet digital PCR data in samples derived from prior drinking water studies, wherein some inexplicable stochastic variations in the *Lp* occurrence were observed in replicate microcosms. We discovered an apparent antagonistic relationship between and . This relationship was noted across three water sources (Flint, Detroit, and Blacksburg) and was at least partially mediated by the presence of copper, through either copper pipes or a dosed range of 0–2000 μg/L total copper. The observations of this study, which was conducted under realistic drinking water conditions harboring mixed microbial communities, are consistent with recent pure culture studies reporting that amoebic uptake may be inhibited when are established as amoebal endosymbionts. The findings may help explain the apparent stochastic behavior of *Lp* in field and research settings and may open a door to new engineered ecological control strategies for *Lp*.

## Graphical abstract

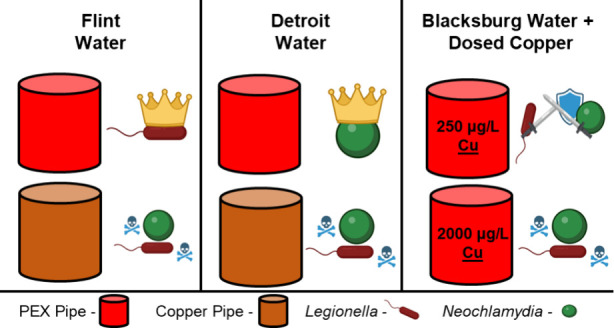

## Introduction

Legionnaires’ disease (LD) is a severe form of pneumonia that is the number one source of tap water associated disease and death in the US,^1^ with incidence also increasing throughout Europe.^2^ LD is caused by various spp., most commonly (*Lp*), which can establish as a member of the drinking water microbial community and infect individuals inhaling contaminated aerosols.^3^ *Lp* is particularly problematic in premise plumbing, where secondary disinfectant residual dosed by the water utility tends to be depleted, the water is warmer and more stagnant, and there is higher surface area for biofilm attachment and growth relative to main distribution system pipes.^4^

Recent microbiome profiling studies using 16S rRNA gene amplicon sequencing have revealed that a typical drinking water sample can contain thousands of microbial species,^5^ with its microbial composition shaped by treatment processes at the drinking water treatment plant, pipe materials, water age, water source characteristics, and other factors.^6–9^ leverage microbial ecological relationships for survival and growth in the oligotrophic drinking water environment,^10^ especially infection of free-living amoebae that graze on biofilm.^11^ Accordingly, a “probiotic” approach to *Lp* control has been proposed, wherein the microbial ecology of the drinking water system could be intentionally manipulated to limit *Lp* proliferation.^12,13^ For example, nonpathogenic bacteria could compete with *Lp* for amoebae hosts or for nutrients. However, such probiotic approaches have remained elusive in practice, with different potential antagonistic bacteria reacting differently to various strains and species of .^14^ Such knowledge gaps must be addressed before a probiotic approach can be fully implemented.^8^

Here we retrospectively examined 16S rRNA gene sequencing data of samples derived from an array of prior drinking water microcosm studies to identify microorganisms that could potentially prevent *Lp* growth. These studies employed replicate microcosms using three distinct drinking waters (Flint, Michigan; Detroit, Michigan; Blacksburg, Virginia) exposed to a range of copper levels arising from either purposeful dosing (0–2,000 μg/L) or pipe material (copper versus PEX).^15,16^

## Materials and Methods

### Overview of Experimental Approach

The two selected studies, referred to herein as the “Flint/Detroit”^15^ and “Copper-Dosing”,^16^ were conducted under comparable conditions. The microcosm design was the same, consisting of 120 mL borosilicate glass bottles (filled with 100 mL bulk water in the Flint/Detroit Study and 110 mL bulk water in the Copper-Dosing study) with various modifications (e.g., addition of Cu/PEX pipes or dosing of soluble copper) and similar water change regimes (2× weekly). For each study, all microcosms contained mature biofilm (>3.5-year-old) and were operated in biological triplicate (*n* = 3), continuously mixed on a shaker table at 100 rpm, and incubated at 37 °C, an optimal growth temperature for *Lp*.^17^

### Flint/Detroit Microcosms

The Flint/Detroit study is detailed in Martin et al.^15^ and SI Table 1. Twelve “Detroit” microcosms were fed municipal water produced by the City of Detroit, which was sourced from Lake Huron and maintained effective corrosion control using orthophosphate. Twenty-four “Flint” microcosms were fed with treated Flint River water, which was collected from the Flint River and then treated in-lab to simulate the municipal water without corrosion control that was produced during the Flint Water Crisis. Half of the microcosms contained copper pipes, and the other half contained PEX pipes, each with an approximate surface area of 85 cm^2^. The microcosm biofilms were initially matured for 7 years prior to this study using dechlorinated Blacksburg tap water.^18–20^ Thereafter, the microcosms were acclimated to either Flint or Detroit water for an additional 1.5 years and subject to 50% volume water changes 2× weekly to simulate infrequent water use. Subsequently, Week 0 of the experiment commenced with inoculation of a mixture of four *Lp* strains, two environmental isolates from a Flint hospital (serogroups 1 and 6), an uncharacterized strain,^15^ and a reference strain (serogroup 1), all mixed proportionally to target 1,000 CFU/mL in each microcosm. Subsets of the Flint microcosms were additionally subject to a range of iron amendments (SI Table 1), which were hypothesized to be an important nutrient source for growth resulting from iron pipe corrosion during the 2014–2016 Flint Legionaries’ disease outbreak.^21^ Microcosms were sampled on Weeks 54 and 55 for culture-based enumeration of *Lp* in bulk water (SI Figure 1) and at Week 60 for DNA analysis of bulk water and biofilm. Difficulty in achieving consistent *Lp* concentrations among some biological replicates was noted in subsequent research using these microcosms.^22^

### Copper-Dosing Microcosms

The Copper-Dosing study is detailed in Smeltz et al.^16^ and in SI Table 2. Fifteen new glass microcosms were equipped with four 2.5 cm length, ∼1.7 cm inner diameter, PEX-b pipes, of which two were new pipe and two were colonized with >3.5-year-old mature biofilm containing *Lp*, which was obtained by cutting portions of the recirculating lines of pilot-scale hot water rigs that were not previously dosed copper.^23,24^ The rigs were inoculated with two *Lp* strains (serogroup 1) that were isolated from Quincy, Illinois during an LD outbreak.^25^

The microcosms were subject to 75% volume water changes twice a week, replacing the bulk water volume with granular activated carbon (GAC) treated local Blacksburg tap water with a final phosphate concentration of 5 μg/L as P. During the first six months of acclimation, the microcosms were subjected to a series of 10 cross-inoculation events to establish a similar baseline. Thereafter, the microcosms were assigned to one of the five experimental conditions, in triplicate, such that there were no significant differences across conditions for *Lp* or total cell counts when copper-dosing commenced. Copper was dosed using CuSO_4_·5H_2_O to achieve 0, 4, 30, 250, or 2000 μg/L total added Cu. During the experiment, *Lp* concentrations began to decline in some replicate microcosms, which prompted efforts to restore the growth across microcosms. This included dosing of ferric pyrophosphate (100 μg as Fe) at 35 weeks to ensure iron was not a limiting nutrient and a follow-up reinoculation of culturable *Lp* of ∼50 MPN/mL to each microcosm, ∼3.5 months into the copper dosing phase, using effluent water from the pilot-scale rig from which the pipes were derived. None of these efforts corrected the divergence of *Lp* concentrations among the 250 μg/L Cu replicates, where one microcosm had consistently higher *Lp* than the other two (SI Figure 1).

After 11 months of copper dosing, GAC-treated influent and the bulk water in each microcosm were sampled for DNA extraction and *Lp* culture during three sequential water changes (3–4 days apart, referred to as three sampling “events” in the captions to Figures 1 and 2). During month 12, two biofilm samples were collected from each microcosm, one from each of the two PEX pipes in each microcosm originally derived from the pilot-scale rig.

**Figure 1:**
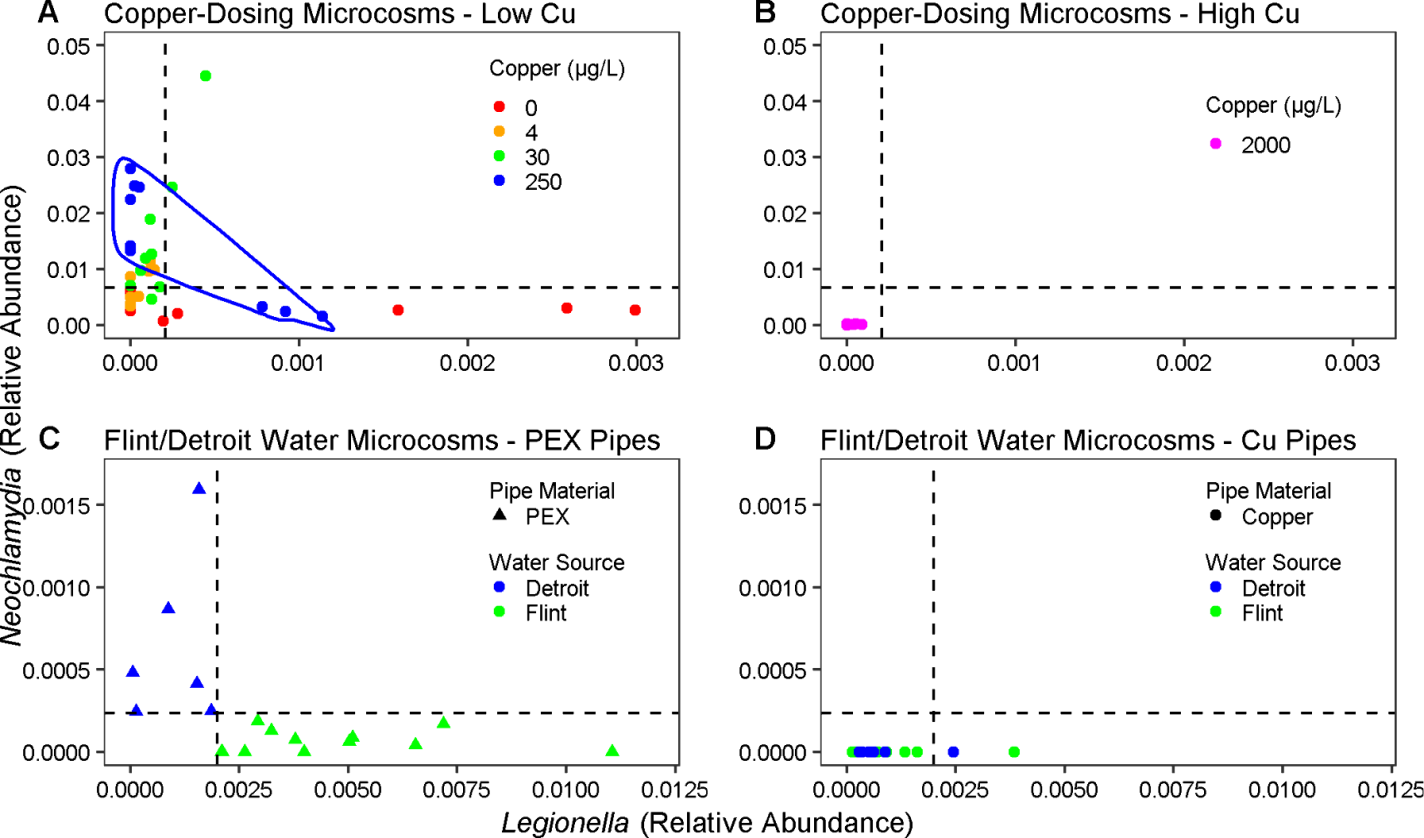
Bulk water vs relative abundance (proportion of total reads per sample) estimated by 16S rRNA gene amplicon sequencing of drinking water microcosms. (A) Copper-Dosing microcosms containing PEX pipes receiving GAC-treated Blacksburg water dosed with 0–250 μg/L Cu [4 copper levels × 3 microcosms × 3 events = 36 data points] or (B) 2,000 μg/L Cu [1 copper level × 3 microcosms × 3 events = 9 data points]. Flint/Detroit microcosms receiving Flint or Detroit water sources (indicated in legend) and containing (C) PEX pipes [Flint (12 microcosms × 1 event = 12 data points) and Detroit (6 microcosms × 1 event = 6 data points)] or (D) copper pipes [Flint microcosms (11 microcosms × 1 event = 11 data points) and Detroit microcosms (6 microcosms × 1 event = 6 data points)]. In (A), the 250 μg/L Cu condition is indicated to highlight divergence among replicates favoring either or . Thresholds for “high” and “low” relative abundance were determined using the average and concentrations in the bulk water and biofilm across the copper-dosing microcosms (A and B), and the Flint/Detroit microcosms (C and D).

**Figure 2:**
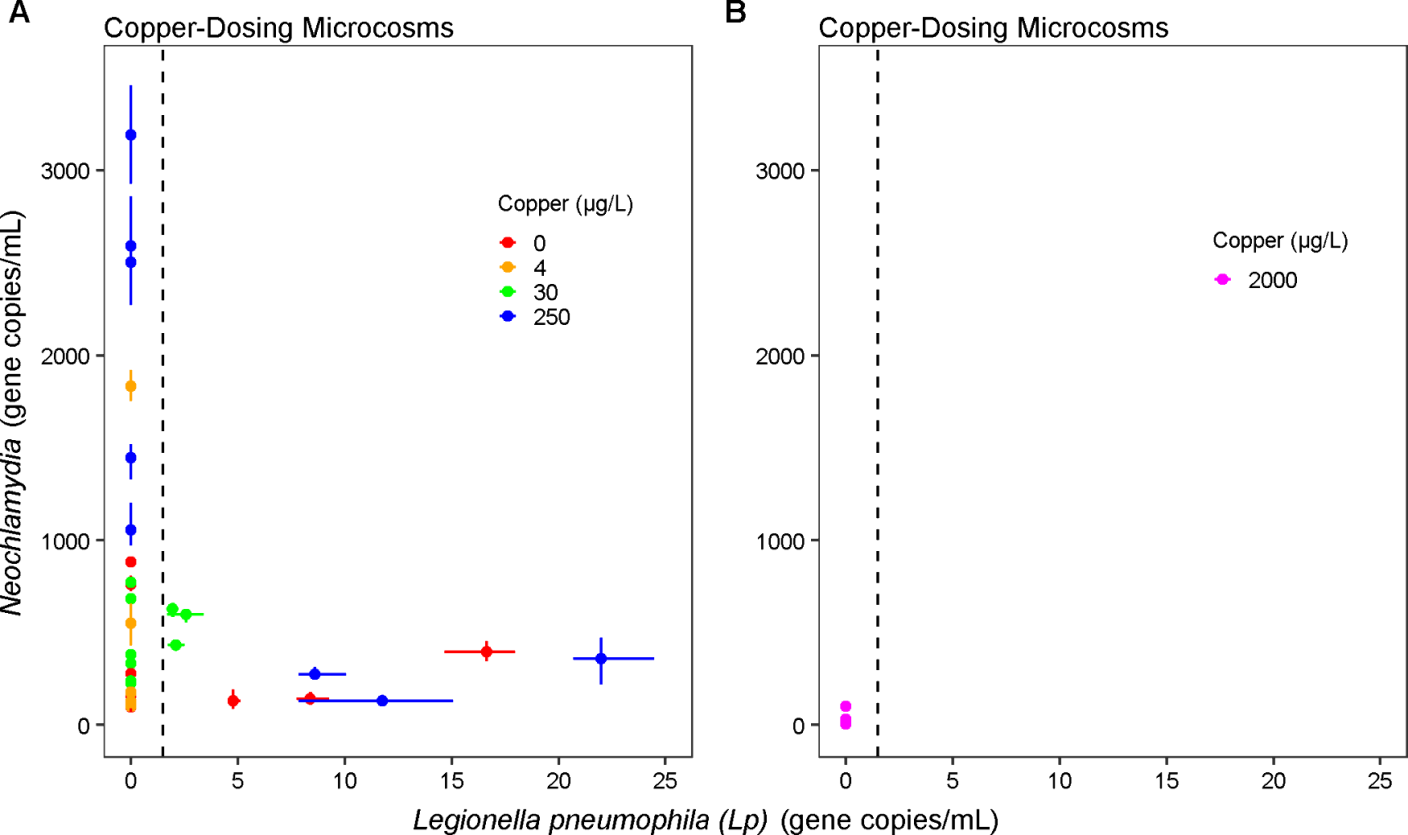
Bulk water *Lp* vs gc/mL in drinking water Copper-Dosing microcosms containing PEX pipes receiving GAC-treated Blacksburg water dosed with (A) 0–250 μg/L Cu [4 copper levels × 3 microcosms x 3 events = 36 data points] or (B) 2,000 μg/L Cu [1 copper level × 3 microcosms × 3 events = 9 data points], measured by ddPCR. The vertical dashed lines represent the estimated detection limit for *Lp*. All samples were detectable for . For *Lp*, points plotted at zero indicate non-detects. Solid lines on each point represent the range interval (lowest and highest) of concentrations for the technical replicates per sample (vertical for and horizontal for *Lp*) whereas points represent the average concentration (among three technical ddPCR replicates).

### Culture-Based Analysis of Legionella, Droplet Digital PCR (ddPCR) Analysis, and Statistical Analysis

Detailed descriptions of the culture methods used in the Flint/Detroit and Copper-Dosing studies, as well as the ddPCR procedures and statistical analyses conducted for this study, are provided in the Supporting Information (SI Text 1, SI Figures 2–5, and SI Tables 3–4). For relative abundance comparisons, thresholds to compare “high” and “low” and relative abundance were set for the Flint/Detroit and Copper-Dosing studies by using the average and abundances across the microcosms in both the biofilm and bulk water.

### Data Availability

16S rRNA sequences are available on the National Center for Biotechnology Information Sequence Read Archive database under Accession numbers PRJNA1242969 (Flint/Detroit) and PRJNA1229645 (Copper-Dosing). Additional data and materials used in this study are available upon request to the corresponding author.

## Results

### Retrospective Analysis Reveals a Possible Antagonistic Relationship Between *Neochlamydia* and *Legionella* in Drinking Water Microcosms

Analysis of the 16S rRNA gene amplicon sequencing data suggested an apparent antagonistic relationship between and across microcosms fed three distinct drinking water types (Figure 1). A threshold-type response was observed in which one or the other organism tended to dominate the other. For instance, in the case of the Flint/Detroit microcosms, there was no case in which relative abundance was >0.0002 when relative abundance was >0.002 (Figure 1c, 1d). Similarly, in the case of the Copper-Dosing microcosms, for the majority of bulk water samples (43/45 collected from the 15 microcosms over three water changes), relative abundance was never >0.0066 when relative abundance was >0.0002 (Figure 1a, 1b).

### Relationship Is Influenced by Copper and Source Water

Copper appeared to influence the relative abundances of and . Among the microcosms with copper pipes that grew , one of each of the Flint and Detroit conditions contained > 0.002 relative abundance, and these microcosms never yielded detectable (Figure 1d). The source water (Lake Huron for “Detroit” and Flint River for the “Flint” microcosms) also played an important role. was frequently detected in the corresponding Flint/Detroit microcosms containing PEX pipe, with all Flint PEX microcosm bulk water levels being higher than all Detroit PEX microcosms. Conversely, the bulk water of all Detroit PEX microcosms contained higher relative abundances than all Flint PEX microcosms (Figure 1c). For the bulk water of the Flint/Detroit microcosms, the log ratio of / relative abundance was not significantly influenced by pipe material (*p* > 0.05, Kruskal–Wallis test), but was significantly influenced by source water (*p* < 0.05, Kruskal–Wallis test).

Among the Copper-Dosing microcosms (which were all fed Blacksburg water), the 0 μg/L Cu condition generally exhibited low and higher compared with the other copper conditions, whereas the 4 μg/L Cu condition generally contained lower levels of both organisms. The 30 μg/L Cu condition was associated with high and low (Figure 1a). At 2,000 μg/L Cu, both and were diminished, consistent with an expected antimicrobial effect of Cu (Figure 1b). The condition with 250 μg/L Cu displayed notable divergence among replicates between the two organisms. One Copper-Dosing replicate microcosm always had high and low relative abundance in triplicate sequential samples, while the other two replicate microcosms always contained high and low relative abundance (Figure 1a). The detected and was attributed to growth in the microcosms, since the influent had undetectable/barely detectable levels of both microorganisms according to 16S rRNA gene amplicon sequencing ( = nondetect; relative abundance = 0.001).

### ddPCR Measurements Are Consistent With the 16S rRNA Gene Amplicon Sequencing Trends

ddPCR was applied and compared with 16S rRNA gene amplicon sequencing trends observed in the Copper-Dosing study, particularly in relation to *Lp* (Figure 2). *Lp* and gc/mL were positively correlated with relative abundances of corresponding genera annotated from amplicon sequencing data (Spearman’s ρ = 0.7, F-test p-value <0.05 for to *Lp*, and Pearson’s r = 0.91, F-test p-value <0.05 for ) (SI Figure 6). ddPCR measurements further aligned with patterns, suggesting a mediating role of copper. *Lp* was below the ∼1.5 gc/mL detection limit in most samples, including the 2,000 μg/L replicates, and at least one replicate in each other copper conditions (with *Lp* being detected in 3 out of 15 microcosms over the course of three sequential water changes, for a total of 9 detections out of 45 samples collected), whereas was always detected across all copper conditions, including 2,000 μg/L (Figure 2). The influent used for water changes had undetectable *Lp* and low (∼2 gc/mL) according to ddPCR, consistent with the 16S rRNA gene amplicon sequencing data.

ddPCR analysis also captured the divergent behavior of and in the replicate microcosms at 250 μg/L Cu (Figure 2a). *Lp* was measured up to 22 gc/mL in one microcosm and was consistently undetectable (<1.5 gc/mL) in the two other microcosms on all 3 days sampled. The two 250 μg/L Cu microcosms with undetectable *Lp* contained very high concentrations of (average ± sd = 2039.6 ± 838.0 gc/mL across the three sampling dates (2 reps × 3 dates)), whereas the replicate with high *Lp* contained low concentrations of (average ± sd = 253.6 ± 115.8 gc/mL across the three sampling dates (1 rep × 3 dates)). The *Lp* ddPCR data also agreed with culturable *Lp* data trends (SI Figure 7) for the Copper-Dosing microcosms (Spearman’s ρ = 0.79, F-test p-value <0.05).

### Comparison of Biofilm and Bulk Water Trends

Occurrence patterns of and in microcosm biofilms generally reflected trends noted in the bulk water (see SI Text 2 and SI Figures 8–10).

### Statistical Analysis with Combined Bulk Water and Biofilm 16S rRNA Gene Amplicon Sequencing Data

For the Copper-Dosing microcosms, pooling together biofilm and bulk water data, the log ratio of to was impacted by copper dosage (*p* < 0.05, *t* test for the copper dose regression coefficient in the linear model; *p* < 0.05, F-test for overall model significance) (SI Table 4). Pooling of data for this analysis was justified by no significant difference in these ratios between the bulk water and biofilm across the copper-dosing microcosms (*p* > 0.05, Kruskal–Wallis).

### Discussion

A retrospective analysis of drinking water microcosm microbiomes provided evidence of a potential antagonistic relationship between *Legionella/Lp* and *Neochlamydia.* This relationship was frequently observed across three distinct drinking waters (Flint or Detroit water for the Flint/Detroit microcosms and Blacksburg water for the Copper-Dosing microcosms) and two pipe materials (copper and PEX). This discovery builds on recent pure culture studies, where certain strains (S13) have been shown to impact *Lp* viability, with the apparent mechanism being influencing phagocytic uptake pathways in amoeba host-cells.^13,26–28^ can also influence the growth rates of both^29^ and^30^ and inhibit amoebae cyst formation^31^ without causing cell lysis,^26^ thus incurring potential indirect effects on amoebae hosts that could also influence *Lp* survival and replication. Our findings suggest that these interactions may have ecological relevance under more realistic drinking water conditions with complex microbiomes. However, we note that the experimental conditions used in this study only focused on 37 °C with little or no disinfectant and infrequent water changes. Future work is needed to determine whether similar trends would occur under a wider range of practically relevant conditions. Therefore, this relationship should be interpreted cautiously and further examined in future studies, especially in a biofilm setting.

This study further suggests that copper may have influenced the outcome of the apparent competition between and in the microcosms (SI Text 3). Copper is of interest as a nutrient and/or antimicrobial that can leach from pipes or be intentionally dosed.^32,33^ The dose of 250 μg/L Cu allowed for a stochastic outcome in which either or tended to be more abundant depending on the replicate. While the copper concentrations tested in this study span a range relevant to premise plumbing systems,^34^ future work should include additional intermediate concentrations and differentiate between total, soluble and free copper to better demarcate impacts on of and .

Based on this study, it is reasonable to assume that microbial ecology could be a fundamental driver of some of the stochasticity commonly observed in other research and field surveys, such as the frequency of positive taps within the same building.^35^ For example, could pre-establish in only a subset of plumbing legs within the same building, within different buildings in the same water distribution system, or in different regions of the same distribution system. Further surveys of and across a wider array of field conditions would be of interest toward harnessing this microbial ecological relationship as a new dimension to mitigate LD transmission risk and disease patterns.

Pipe material also influenced the two organisms. With a PEX pipe, preferred Detroit water, while preferred Flint water. In the Flint/Detroit microcosms, copper pipe generally acted to suppress , providing the opportunity for to be elevated in bulk water under some conditions. For the microcosms studied herein, was more successful at colonizing biofilm of Cu pipes, especially when fed the more corrosive Flint water, which frequently released higher levels of soluble and total copper into the water than Detroit water.^15^ Given that the soluble Cu concentrations in the microcosms with Cu pipe in the Flint/Detroit study were in the range of 130–950 μg/L (when comparing the mean concentrations from each condition) (SI Table 5),^15^ this suggests that there are additional dimensions to how soluble vs metallic copper influence and that are worthy of additional investigation. Nonetheless, this discovery could help explain unusual situations in which is sometimes found at much higher levels in situations with copper versus plastic plumbing.^36,37^

In the Flint/Detroit microcosms, the relative dominance of versus depended on the water source. The Flint microcosms that were fed Flint River water with high levels of organic carbon and associated with a major LD outbreak^21^ supported persistent and minimal . Conversely, microcosms fed Detroit water (originating from Lake Huron) were colonized by abundant and minimal , which is consistent with lower incidence of LD before and after the Flint Water Crisis when using this water source.^21^ The overall patterns suggest that chemical or microbial differences in source water may dictate which organism successfully colonizes and persists in premise plumbing environments. Future research could aim to further delineate the role of various microbiological and chemical characteristics of the drinking water source in influencing whether or dominates.

The present study provides compelling evidence that may play an unrecognized ecological role that influences in a range of drinking water chemistries and pipe materials. At present, there is no evidence that spp. are human pathogens, but other members of the family Parachlamydiaceae have recently been identified as potential infectious agents in 1–2% of lower respiratory tract infections.^38^ In any effort to advance a true “probiotic” addition of microbes to drinking water with the intention of controlling or other pathogens, it would ultimately be important to confirm the safety of the approach. Our work suggests this apparent antagonistic relationship is already occurring naturally in some situations.

Due to the retrospective nature of this study using the limited DNA available from archived samples, we were unable to quantify amoeba host cells or total bacterial abundance. Future work could include such measurements, employing a wider range of water sources and a greater number of replicates to elucidate the ecological role of and the overall microbial context in dictating persistence and growth.

## Supplementary Material



## Notes

Additional information regarding the Flint/Detroit and Copper-Dosing studies (experimental conditions and culturable *Lp* data with methods used), ddPCR assays, ddPCR and statistical analysis used in this study, differential abundance analysis of the 250 μg/L Copper-Dosing microcosms, in-lab validation of the assay, comparisons between ddPCR/16S rRNA gene amplicon sequencing/culturing results, extensive biofilm results, and additional comparisons between the biofilm and bulk water trends (PDF)
