# Supplementary material for: Retrospective Analysis of Drinking Water Microcosm Microbiomes Reveals an Apparent Antagonistic Relationship between and
Source: Environ Sci Technol Lett. 2025 Jul 17;12(8):990–6. doi: 10.1021/acs.estlett.5c00590 (PMC12351522; doi:10.1021/acs.estlett.5c00590)
Supplement: Supplementary file 1 [file ez5c00590_si_001.pdf]

Supporting Information for:

Retrospective Analysis of Drinking Water Microcosm Microbiomes Reveals an Apparent Antagonistic Relationship Between *Neochlamydia* and *Legionella*

Authors:

Fernando A. Roman Jr[‡1], Thomas Byrne[‡2], Rebekah L. Martin[3], Didier Mena-Aguilar[4], Rania E. Smeltz[1,5], Rachel Finkelstein[1,6], Amy Pruden[1], Marc A. Edwards*[1]

[1]Department of Civil and Environmental Engineering, Virginia Tech, Blacksburg, Virginia 24061, United States

[2]Department of Genetics, Bioinformatics, and Computational Biology, Virginia Tech, Blacksburg, Virginia 24061, United States

[3]Department of Civil and Environmental Engineering, Virginia Military Institute, Lexington, Virginia, 24450, United States

[4]Department of Biochemistry, University of Nebraska-Lincoln, N106, The Beadle Center, Lincoln, Nebraska 68588-0664, United States

[5]Department of Microbiology, University of Alabama at Birmingham, Birmingham, Alabama 35294-2170, United States

[6]AECOM, 3101 Wilson Boulevard, Arlington, Virginia 22201, United States

[‡]These two authors are designated as co-first authors and contributed equally to this work

*Corresponding Author edwardsm@vt.edu

Contents:

Supporting Information Text 1. Culture-Based Analysis of *Legionella*, Droplet Digital PCR (ddPCR) Analysis, and Statistical Analysis used.

Supporting Information Text 2. Biofilm results (and comparison to bulk water trends) for the Flint/Detroit and Copper-Dosing microcosms

Supporting Information Text 3. Expanded discussion on the influence of copper on the microcosms

Supporting Information Table 1: Additional information for Flint/Detroit study

Supporting Information Table 2: Additional information for Copper-Dosing study

Supporting Information Table 3: ddPCR assays used in study

Supporting Information Table 4: Regression analysis on Copper-Dosing microcosms

Supporting Information Table 5: Copper concentrations in Flint/Detroit microcosms containing copper pipes

Supporting Information Figure 1: Culturable *Lp* data from Flint/Detroit and Copper-Dosing studies

Supporting Information Figure 2: Differential abundance analysis at the genus level for the 250 µg/L replicates

Supporting Information Figure 3: *Neochlamydia* assay in lab validation

Supporting Information Figure 4: Log of *Neochlamydia* to *Legionella* for the Copper-Dosing microcosms

Supporting Information Figure 5: Log of *Neochlamydia* to *Legionella* for the Flint/Detroit microcosms

Supporting Information Figure 6: 16S rRNA Relative abundance comparison to ddPCR results for *Neochlamydia* and *Legionella/Lp*

Supporting Information Figure 7: *Neochlamydia* relative abundance versus Bulk water culturable *Lp* in Copper-Dosing study

Supporting Information Figure 8: *Neochlamydia* and *Legionella* relative abundance in bulk water versus biofilm for Flint/Detroit study

Supporting Information Figure 9: *Neochlamydia* and *Legionella* relative abundance in bulk water versus biofilm for Copper-Dosing study

Supporting Information Figure 10: *Neochlamydia* and *Legionella* relative abundance within Flint/Detroit and Copper-Dosing microcosm biofilms.

Supporting Information Text 1.

Culture-Based Analysis of Legionella

Flint/Detroit samples were subject to culture-based enumeration of viable CFU *Lp*/mL by plating on GVPC buffered charcoal yeast extract agar (ISO 11731)[1], with a subset of colonies from each microcosm confirmed as *Lp* via PCR.[2] The Copper-Dosing samples were analyzed using the non-potable Legiolert™ (IDEXX Laboratories, Westbrook, ME, USA) procedure as MPN/mL.[3]

DNA Extraction, 16S rRNA Gene Amplicon Sequencing and Data Analysis

Bulk water samples of defined volumes were concentrated onto 0.22-µm mixed-cellulose ester membrane filters (Cenmed Enterprises, New Brunswick, NJ, USA[2] or MilliporeSigma, Burlington, MA, USA)[3]. Biofilms were swabbed from the interior of pipes in all the microcosms, using either polyester[2] (Cardinal Health, Dublin, OH, USA) or cotton-tipped swabs[3] (Puritan Medical Products, Guilford, ME, USA). Filters and swab tips were transferred to 2-mL Lysing Matrix A tubes. DNA was extracted from filters and swabs using the bacterial protocol of the FastDNA SPIN kit (MP Biomedicals, Solon, OH, USA). 16S rRNA genes were subject to amplicon sequencing via Illumina MiSeq (Illumina, San Diego, CA, USA), using the 515F-926R primer pair, targeting the V4-V5 hypervariable regions, as described in the corresponding studies.[3,4]

16S rRNA amplicon sequence reads were demultiplexed and quality filtered using Qiime2 version 2020.6[5]. Reads were denoised with DADA2[6] and amplicon sequence variants were aligned. Taxonomy was assigned to variants using the naïve Bayes q2-feature-classifier[7] against the SILVA 138.2 99% OTUs reference sequences[8]. All further analysis and statistics were conducted in R (version 4.2.2)[9,10] at the genus taxonomic level. Total sum scaling was used to normalize genus proportions to sample sequencing depth, accounting for sequencing depth variation.[11,12] Differential abundance analysis and Wald significance tests were conducted to compare divergent replicates using the DESeq2 package[13] with reads rarefied to the lowest common sequencing depth (6,842 for the Copper-Dosing microcosms, and 10,017 for the Flint/Detroit microcosms) using the vegan package (version 2.6-4)[14] (SI Figure 2).

Droplet Digital PCR (ddPCR) Analysis

A QX200 droplet digital PCR (ddPCR) (Bio-Rad, Hercules, CA, USA) was used to quantify *Lp* (targeting the *Lp* specific *mip* gene)[15] and *Neochlamydia* (targeting the 16S rRNA gene)[16] gene copy (gc) numbers using probe-based assays subject to in-house validation for target specificity in the microcosm environment (SI Table 3, SI Figure 3). For both assays, all samples were run in technical triplicate, with a positive detection threshold of ≥3 positive droplets[17] in at least two out of three of the technical replicates. Each well had a total reaction volume of 22 µL, with 20 µL used for analysis. Final reactions contained 250 nM probe, 900 nM of each primer, and were prepared using ddPCR Supermix for probes (no dUTP) (Bio-Rad). PCR amplification was performed using a C100 Touch Thermal Cycler (Bio-Rad), with thermocycling conditions detailed in SI Table 3. Each 96-well plate contained three no template controls (NTC) (molecular grade water). Additionally, each plate contained three positive control

wells consisting of synthetic gene fragments (gBlocks^{TM}, Integrated DNA Technologies, Coralville, IA, USA) containing the *mip* gene region targeted by the *Lp* assay and the 16S rRNA gene region targeted by the *Neochlamydia* assay. Only wells with > 10,000 accepted droplets were included in the final analysis.

<u>*Statistical Analysis*</u>

Kruskal–Wallis tests were conducted on the log of the ratios of *Neochlamydia*/*Legionella* relative abundance (log_{10}[*Neochlamydia* relative abundance/*Legionella* relative abundance]) (SI Figures 4 and 5) due to the data being non-normally distributed ($p < 0.05$, Shapiro-Wilk test), to assess effects of various metadata. Bidirectional stepwise regression was conducted on the log of the ratio of *Neochlamydia/Legionella* relative abundance (Copper-Dosing microcosms) using stepAIC and a Bayesian Information Criterion to penalize complexity (MASS package version 7.3.61)[18]. An F-test was conducted on the regression model to assess the significance of copper dose (SI Figure 4, SI Table 4, Copper-Dosing microcosms). Individual coefficients in the regression model were assessed for significance using a t-test (SI Table 4). For each correlation analysis, the data was log-log transformed and tested for normality using the Shapiro-Wilk test. Depending on the distribution, either Pearson's or Spearman's correlation coefficient was computed, a linear model was fit, and an F-test was used to assess the overall significance of the model fit (SI Figures 6, 7, 8, and 9). For statistical correlation analysis, nondetects with ddPCR and Legiolert^{TM} were set to their estimated limit of detection: 1.5 gc/mL for ddPCR and 1 MPN/mL for Legiolert^{TM} (using the non-potable 1 mL protocol). For the Copper-Dosing microcosms, repeated measurements (i.e., the three sequential bulk water samples and the two biofilm swabs per microcosm) were averaged as technical replicates to represent each individual microcosm.

Supporting Information Text 2.

Occurrence patterns of *Legionella* and *Neochlamydia* in microcosm biofilms generally reflected trends noted in the bulk water. *Neochlamydia* relative abundance correlated positively in the biofilm versus bulk water [Flint/Detroit Spearman's $\rho = 0.86$, F-test $p < 0.05$ (SI Figure 8a); Copper-Dosing Spearman's $\rho = 0.85$, F-test $p < 0.05$ (SI Figure 9a)]. However, there was a notably weaker or no correlation between relative abundances of *Legionella* in the biofilm vs bulk water [Flint/Detroit Spearman's $\rho = 0.1$, F-test $p > 0.05$ (SI Figure 8b); Copper-Dosing Spearman's $\rho = 0.75$, F-test $p < 0.05$ (SI Figure 9b)].

In the Copper-Dosing experiments, the two organisms displayed the same divergent occurrence patterns in biofilm that was noted above in the analysis of bulk water, including divergence among the biological replicates at 250 µg/L Cu (SI Figure 10a). The biofilm of these microcosms was also found to be depleted in both *Neochlamydia* and *Legionella* in the 2000 µg/L Cu condition (SI Figure 10b).

In all microcosms containing PEX pipes and fed Detroit water (6/6), the biofilms that bore high *Neochlamydia* (>0.0002 relative abundance) were also characterized by low *Legionella* (<0.002 relative abundance) (SI Figure 10c) relative to each organism within the microcosms. In all microcosms containing copper pipe (17/17), *Neochlamydia* was undetectable (SI Figure 10d). The microcosms containing copper pipes and fed Detroit water had bore similar trends in *Legionella* between the biofilm and bulk water, with 6/6 biofilm samples and 5/6 bulk water samples having low *Legionella*. The copper pipes fed with Flint water differed in that most biofilm samples (9/11) contained high levels of *Legionella*, whereas under the same conditions, the majority of bulk water samples (10/11) had low *Legionella* levels (Figure 1d, SI Figure 10d). The microcosms containing PEX pipe and fed Flint water are also a case where biofilm and bulk water trends differed. In the bulk water, all PEX samples (12/12) contained high *Legionella*. and low *Neochlamydia* (Figure 1c), whereas in the biofilm, only one PEX sample contained high *Legionella*, and 9/12 samples contained high *Neochlamydia* (SI Figure 10c). For the biofilm of the Flint/Detroit microcosms, the log ratio of *Neochlamydia*/*Legionella* relative abundance was significantly influenced by pipe material and source water ($p < 0.05$, Kruskal-Wallis tests).

Supporting Information Text 3.

With the 0 µg/L added copper condition, there was low relative abundance and gc/mL for *Legionella*, *Lp*, and *Neochlamydia*, compared to some of conditions with added copper (with the exception of one 0 µg/L Cu replicate which had high *Lp*/*Legionella)*. However, at low doses of 4 or 30 µg/L total Cu, *Neochlamydia* tended to be more relatively abundant compared to *Legionella*. Furthermore, both organisms were controlled at a high dose of 2,000 µg/L. However, 250 µg/L was a case in which the two organisms bifurcated in their relative abundance among the three replicate microcosms. The analysis here provides a plausible answer as to how one of the three replicates persistently maintained high levels of *Lp* and low *Neochlamydia*, whereas the other two maintained low levels of *Lp* and high *Neochlamydia,* despite numerous efforts to rectify differences via inoculation and nutrient adjustment.[3]

Supporting Information Table 1. Conditions and number of samples in the Flint/Detroit drinking water study described in Martin et al. 2022[2]. The microcosms (containing either PEX or copper pipes) had been aged, with consistent water changes (2-3× per week) for 7 years prior to this experiment. Then, for an additional 1.5 years with either Flint or Detroit water, with water changes 2× a week, prior to the tested experimental conditions. After the 9.5 years of aging the microcosms, *Lp* was inoculated into each microcosm so that each achieved 1000 CFU/mL of *Lp*, where differences in iron additions were tested for 60 weeks. Microcosms were dosed with 300 µg/L total iron of either ferric chloride, ferrous chloride, ferric pyrophosphate, and controls with no iron. Originally, 36 microcosms were utilized, however, one replicate microcosm (a Flint water source, no iron added copper condition microcosm) broke by the end of the experiment. Each microcosm was created using 120 mL glass jars, 85 cm^2 of pipe material, and contained 100 mL bulk water.

Water Source	Iron Addition	Pipe Material + Number of Replicates	
Filtered Treated Flint River Water	None	PEX (3)	Copper (2)
	Ferric Pyrophosphate (300 µg/L)	PEX (3)	Copper (3)
	Ferric Chloride (300 µg/L)	PEX (3)	Copper (3)
	Ferrous Chloride (300 µg/L)	PEX (3)	Copper (3)
Filtered Detroit Tap Water (Corrosion Control)	None	PEX (3)	Copper (3)
	Ferric Pyrophosphate (300 µg/L)	PEX (3)	Copper (3)

Supporting Information Table 2. Conditions and number of samples in Copper-Dosing study detailed in Smeltz et al. 2025[3]. After a 6-month acclimation period establishing the fifteen microcosms, the microcosms were separated into five groups of triplicates, where each group was dosed a different amount of copper (dosed as $CuSO_4$) ranging from 0-2000 µg/L (total Cu), for a copper-dosing period of 11 months. Each microcosm was created using 120 mL glass jars, four 2.5 cm length PEX pipes (two of which contained >3.5-year-old mature biofilm), and filled with 110 mL of bulk water. The microcosms were also subject to water changes 2× per week.

**Water Source**	**Copper Addition (Total Cu)**	**Pipe Material + Number of Replicates**
GAC Treated Blacksburg Water	0 µg/L	PEX (3)
	4 µg/L	PEX (3)
	30 µg/L	PEX (3)
	250 µg/L	PEX (3)
	2000 µg/L	PEX (3)

Supporting Information Table 3. Summary of ddPCR assays used with additional information.

Target	Primers/Probes	Annealing Temperature (°C)	Amplicon Size (bp)	Reference	In-Lab Validation
L. pneumophila	Forward: AAAGGCATGCAAGACGCTATG	58.9	78	(Nazarian et al., 2008)[15]	(Wang et al. 2012)[19]
	Reverse: GAAACTTGTTAAGAACGTCTTTCATTTG				
	Probe: FAM-TGGCGCTCAATTGGCTTTAACCGA				
Neochlamydia	Forward: CTCGTGCCGTGAGGTGTT	58.1	167	(Vouga et al., 2015)[16] Based on Sequence: EU683885.1	(Figure S3)
	Reverse: AGCACGTGTGTAGCCCCA				
	Probe*: FAM-TTGGGTGGGAACTCTAATGAGACTGCCT				

PCR amplification for both assays was carried out using the following thermal cycling protocol: **Enzyme Activation** at 95°C for 10 minutes, 40 cycles of **Denaturation** at 94°C for 30 seconds, 40 cycles of **Annealing** (at 58.9°C for *L. pneumophila*, and 58.1°C for *Neochlamydia*) for 1 minute, an **Enzyme Deactivation** of 98°C for 10 minutes, then a hold at 4°C. Both assays had previously undergone in-lab thermal gradient tests to determine the optimal annealing temperature for negative and positive droplet separation.

For the Copper-Dosing study, to assess potential PCR inhibition, all samples were initially run using three dilutions: undiluted, 1:10, and 1:100. For *Lp*, concentrations across the microcosms were generally low, so undiluted samples were used for final quantification. For *Neochlamydia* however, most samples in the Copper-Dosing study (42 out of 45 total, covering 15 microcosms over 3 different days) were quantified using a 1:10 dilution (except for 3 samples from the 2000 μg/L Cu condition which had to be run undiluted due to low *Neochlamydia* signal in those samples).

***Note:** The probe sequence used for *Neochlamydia* (T**TG**GGTGGGAACTCTAATGAGACTGCCT) differs by two base pairs from the probe reported in Vouga et al. (2015)[16], which reported the corresponding base pairs as **CA**. Upon reviewing the original target sequence (EU683885.1)[20], the version reported in this study was found to avoid mismatches. The discrepancy was confirmed in personal correspondence with Dr. Gilbert Greub, the corresponding author of the original publication, who recommended using this corrected primer sequence.

Supporting Information Table 4. Summary of the linear model predicting the log ratio of *Neochlamydia* to *Legionella* 16S rRNA amplicon sequencing relative abundance for the Copper-Dosing microcosms. The full model included the variables of copper dose (both linear and as a polynomial were tested) and sampling medium (biofilm and bulk water). In R, the full model equation is $\log_{10}$(*Neochlamydia*/*Legionella*) ~ [sampling medium] × [(copper dose) + (copper dose)^2]. The final model equation only includes copper dosage as a linear term: in R being $\log_{10}$(*Neochlamydia*/*Legionella*) ~ [copper dose]. F-test for overall model significance gives a p-value of 0.0004905. Adjusted R-squared is 0.334. Model built using combined biofilm and bulk water Copper-Dosing data. The model was applied using 15 bulk water values (averaged from three sampling events per microcosm) and 15 biofilm values (averaged from two bioswabs per microcosm), so that each value represented a single microcosm from the Copper-Dosing study**.**

	Estimate	Standard Error	t-value	p-value
(Intercept)	2.1256917	0.1756480	12.102	1.22e-12
Copper	-0.0007681	0.0001948	-3.942	0.000491

Supporting Information Table 5. Effluent (mean ± standard deviation) bulk water copper concentrations in Flint/Detroit drinking water microcosms containing copper pipes, described in Martin et al. 2022[2].

**Water Source**	**Iron Addition**	**Total Effluent Copper (µg/L)**	**Soluble Effluent Copper (µg/L)**
Filtered Treated Flint River Water	None	1,000 ± 300	880 ± 310
	Ferric Pyrophosphate (300 µg/L)	1,900 ± 860	680 ± 140
	Ferric Chloride (300 µg/L)	2,200 ± 830	680 ± 220
	Ferrous Chloride (300 µg/L)	2,300 ± 800	950 ± 300
Filtered Detroit Tap Water (Corrosion Control)	None	1,300 ± 870	130 ± 74
	Ferric Pyrophosphate (300 µg/L)	1,300 ± 440	130 ± 64

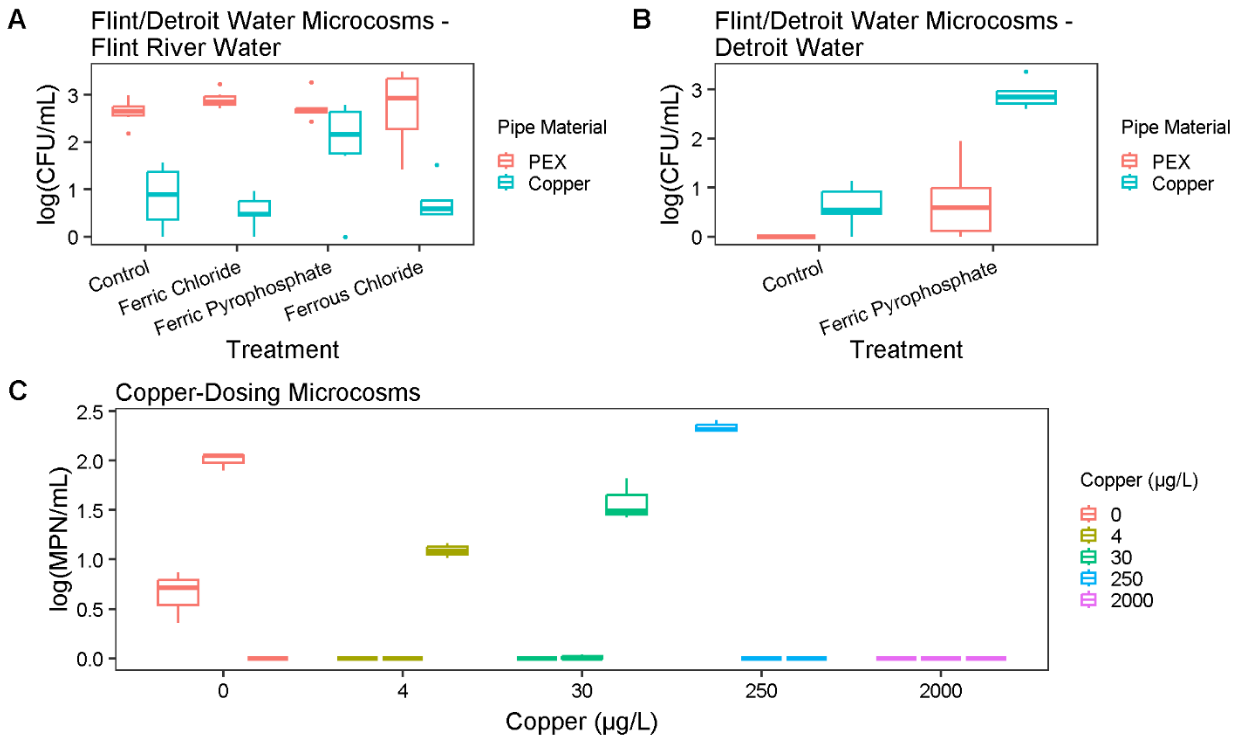

Supporting Information Figure 1. Culturing of *Lp* (A, B) on BCYE GVPC agar plates and (C) with Legiolert™ trays. Log colony forming units/mL for the Flint/Detroit study are shown by corrosion treatment and pipe material for (A) Flint River source water (2 iron conditions, 2 pipe materials, 3 microcosms and 2 measurements - 1 broken microcosm = 46 data points and (B) Detroit source water replicates (2 iron conditions, 2 pipe materials, 3 biological replicates, and 2 measurements = 24 data points), which was cultured on two different dates (weeks 54 and 55 of the study) with a detection limit of 0.01 CFU/mL. Legiolert™ log most probable number/mL is shown by copper concentration and replicate in the (C) Quincy strain study, with each replicate cultured on three different dates (3-4 days apart) on the 11 month of the Copper-Dosing experiment, with replicates shown as indicated in the corresponding boxplot (5 copper levels × 3 microcosms × 3 events = 45 data points) (and a non-potable Legiolert™ detection limit of 1 MPN/mL). Data are compiled and re-analyzed from Martin et al. 2022[2] and Smeltz et al. 2025[3].

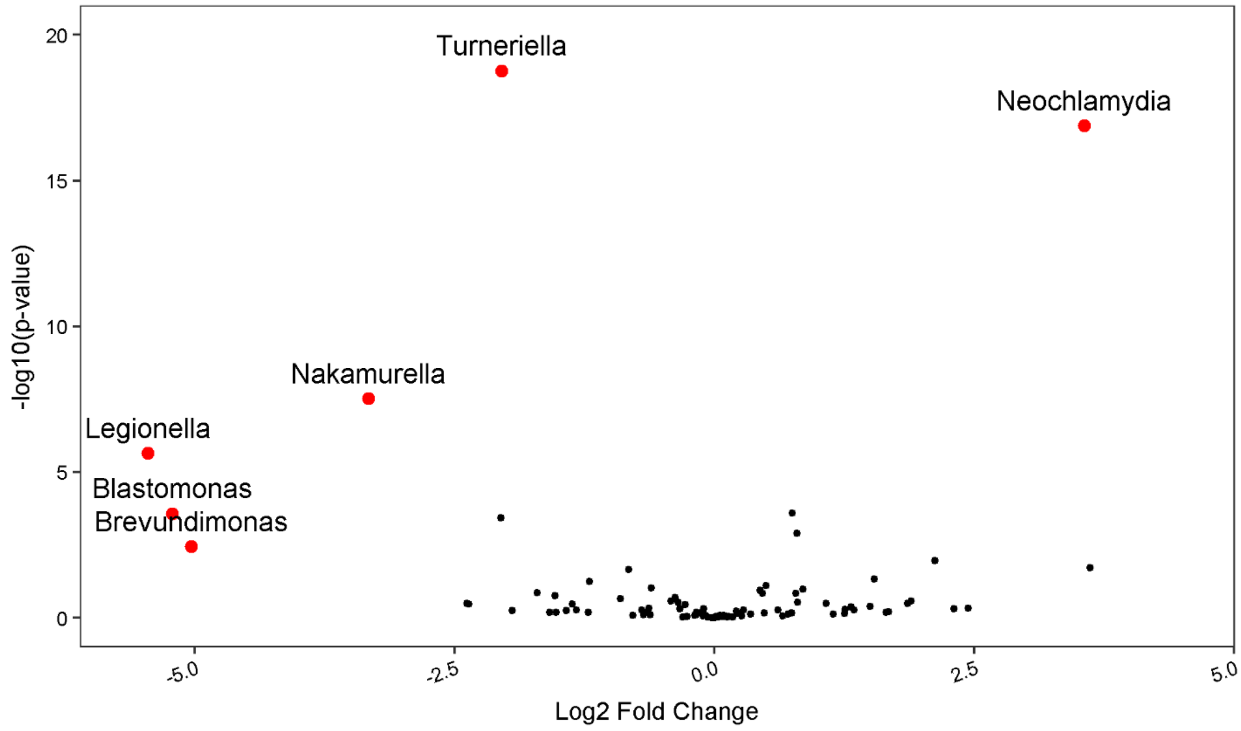

Supporting Information Figure 2. Log fold difference among taxa at the genus level in rarefied 16S amplicon sequencing libraries, between the three sampling dates of two 250 µg/L Copper-Dosing microcosms with high *Lp* as found with ddPCR against the one 250 µg/L Copper-Dosing microcosm with no *Lp* as found with ddPCR. Significantly different taxa as measured by Wald significance tests are shown on the y-axis. Made using 1 copper level × 3 microcosms × 3 events = 9 data points (3 from each microcosm in the 250 µg/L condition).

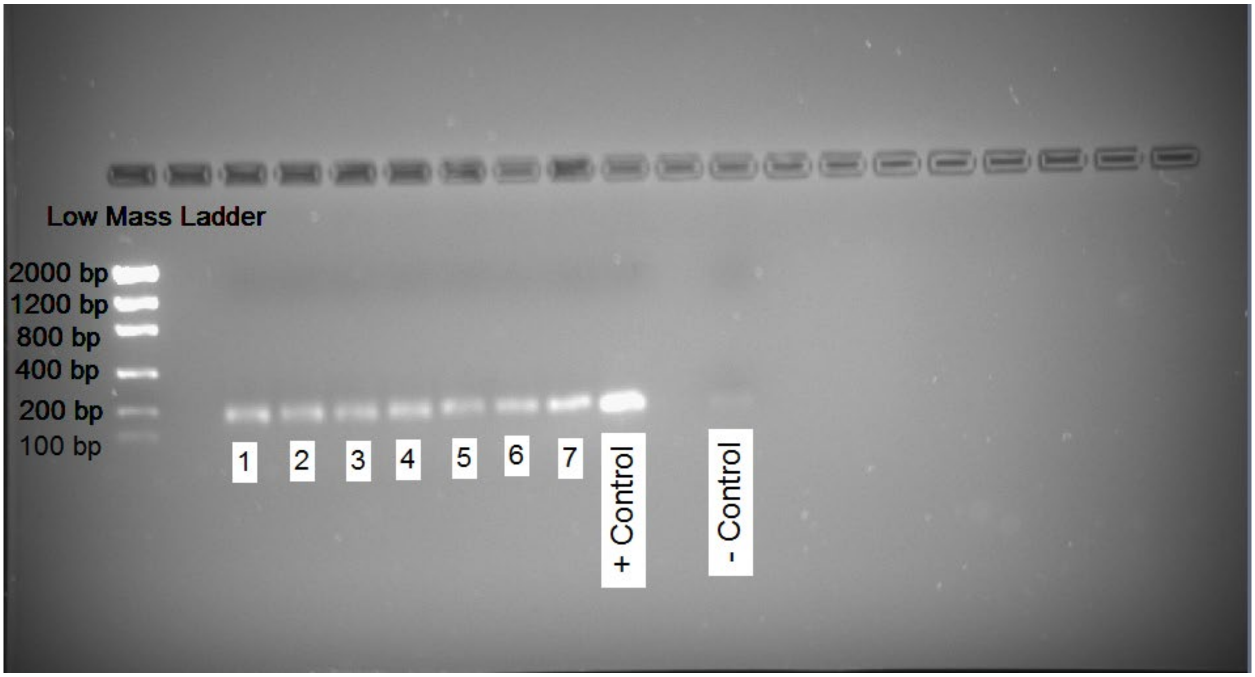

Supporting Information Figure 3. The *Neochlamydia* assay was validated by verifying the expected size of the product (167 bp) with a 2% agarose gel, and SYBR Safe DNA Gel Stain Green (Invitrogen, Waltham, MA, USA), from the product/substrate of a q-PCR run that used the forward/reverse/probe for the *Neochlamydia* target using 7 samples from the Copper-Dosing experiment that contained *Neochlamydia* according to 16S rRNA gene amplicon sequencing (1-7), gBlock/positive control, and a no added template negative control. This gel validated the *Neochlamydia* assay, with a lack of smearing, and a well-defined single band of amplified PCR product, between 100 and 200 bp, compared to the Low DNA Mass Ladder (Invitrogen, Waltham, MA, USA).The resulting q-PCR product was subject to cleaning via a QIAquick PCR purification kit (Qiagen, Venlo, Netherlands) and submitted for Sanger sequencing with the forward primer and processed using an 3730xl DNA Analyzer (Applied Biosystems, Foster City, CA,USA). The resulting Sanger sequence (trimmed based on quality scores) matched the expected region of the *Neochlamydia* target sequence (EU683885.1)[20], including alignment with the reverse complement and probe regions. Additionally, both the *Neochlamydia* and *Lp* probe-based assays were previously subject to thermal gradients ranging from 50 to 65°C within our lab to identify their optimal annealing temperatures. It is expected that the probe would further enhance specificity of the assay, but this cannot be assessed via gel or DNA sequence analysis.

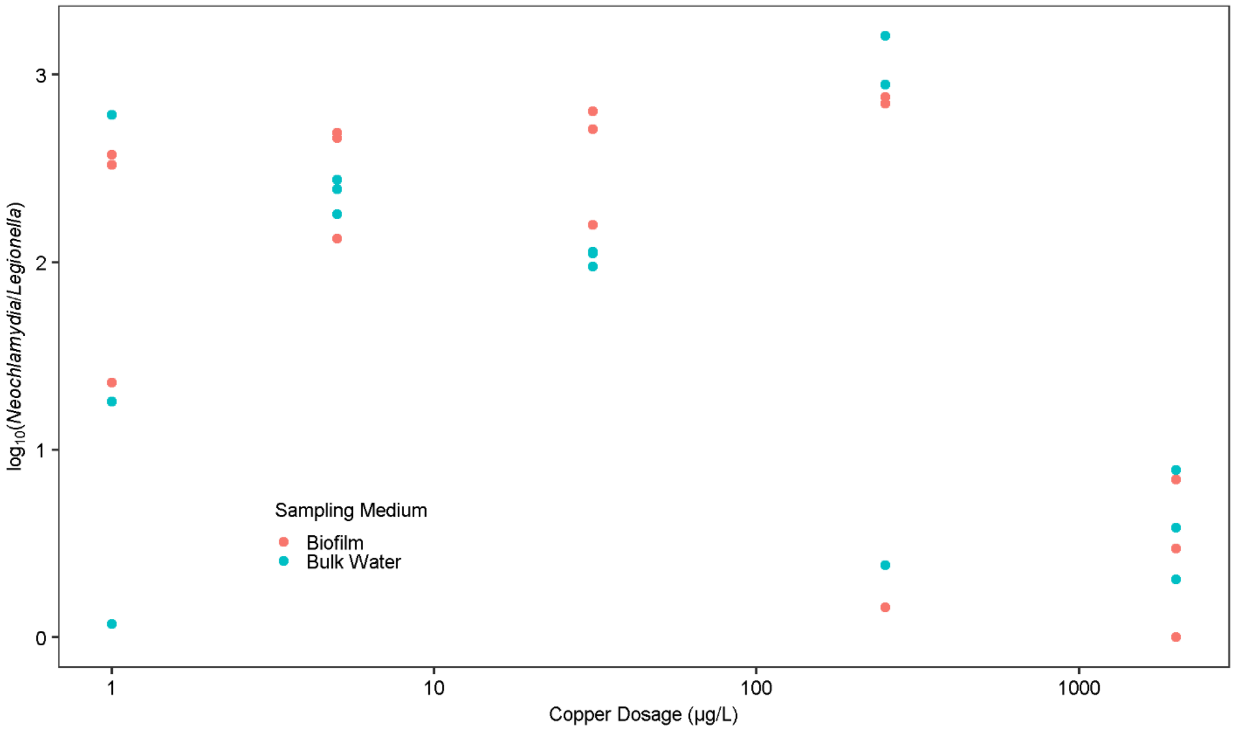

Supporting Information Figure 4. Log ratio ($\log_{10}$ *Neochlamydia*/*Legionella*) of 16S rRNA amplicon sequencing relative abundance across copper dosages for the Copper-Dosing microcosms color-coded by sampling medium. Where 0.0001 is added to each relative abundance to avoid log transforming 0. Copper dosage at 0 is increased by 1 to allow the x axis to be viewed on a log scale for aesthetic purposes. Figure includes 90 data points both bulk water (n=15) and biofilm (n=15) copper dosing data. For bulk water, data were derived from 5 copper levels × 3 microcosms × 3 sampling events = 45 samples, which were averaged by event to yield 15 data points (one per microcosm). For biofilm, 5 copper levels × 3 microcosms × 2 swabs = 30 swab samples, which were averaged to 15 data points (one per microcosm).

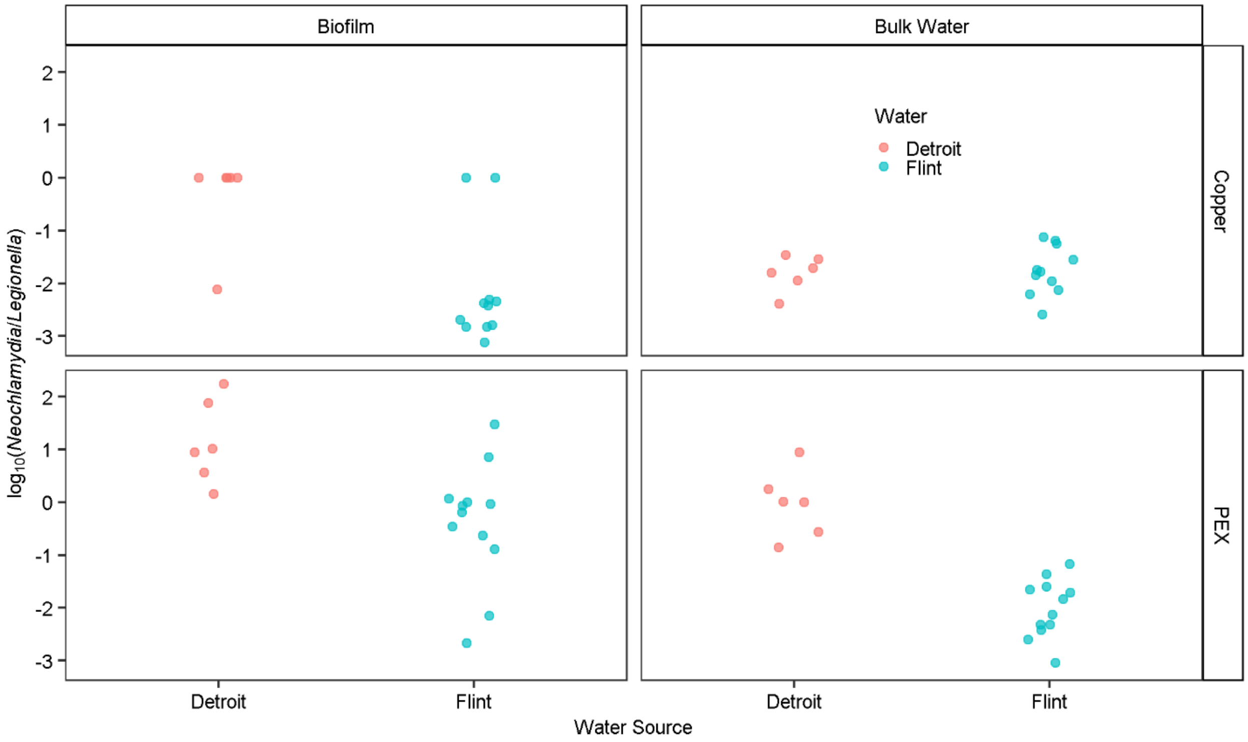

Supporting Information Figure 5. Log ratio ($\log_{10}$ *Neochlamydia*/*Legionella*) of 16S rRNA amplicon sequencing relative abundance across water source, pipe material, and sampling medium for the Flint/Detroit microcosms. Where 0.0001 is added to each relative abundance to avoid log transforming 0. For bulk water, data were derived from 35 microcosms × 1 event (one per microcosm). For biofilm, data were also derived from 35 microcosms × 1 event (one per microcosm).

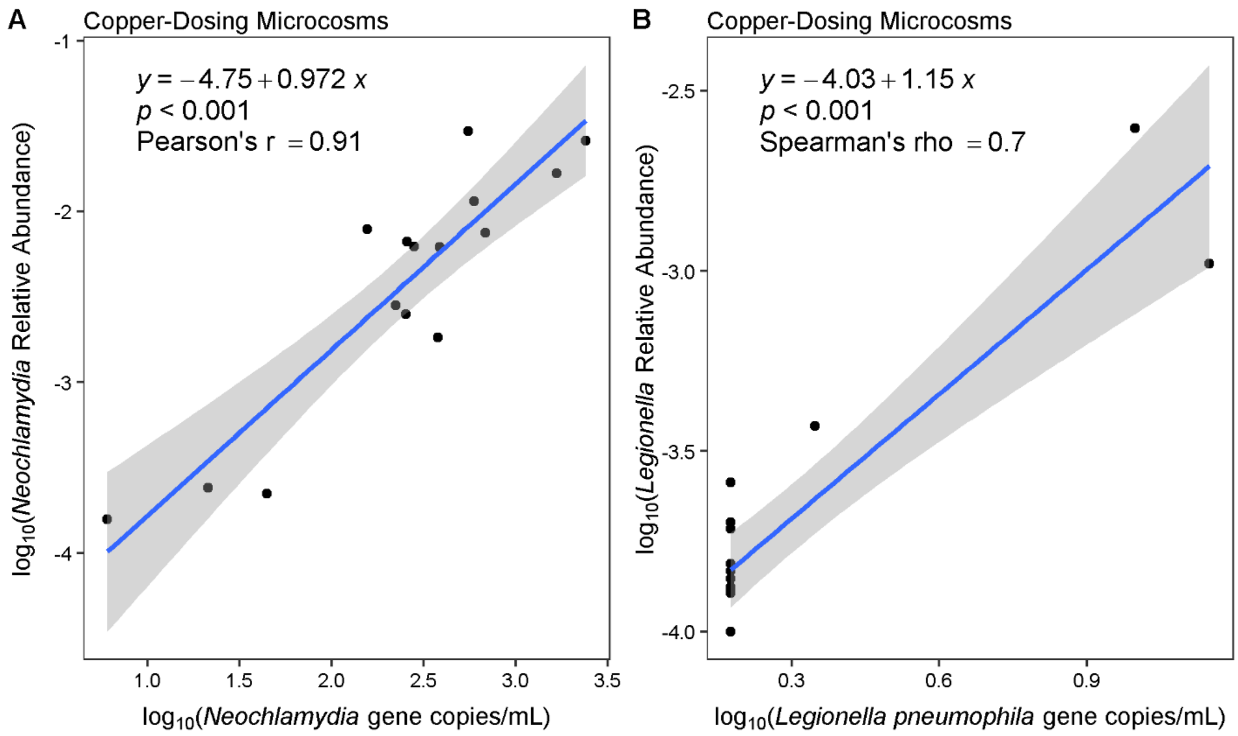

Supporting Information Figure 6. Log transformed comparison of (A) *Neochlamydia* and (B) *Legionella pneumophila* concentrations (gene copies/mL) using ddPCR with (A) *Neochlamydia* and (B) *Legionella* relative abundance as detected through 16S rRNA gene sequencing in the Copper-Dosing microcosms. Where 0.0001 is added to each relative abundance to avoid log transforming 0. ddPCR values represent the average concentration amongst technical triplicates. Both datasets [ddPCR (gc/mL) and 16S rRNA sequencing (relative abundance)] include 5 copper levels × 3 microcosms × 3 sampling events = 45 measurements, which were averaged across events to yield 15 data points (one per microcosm).

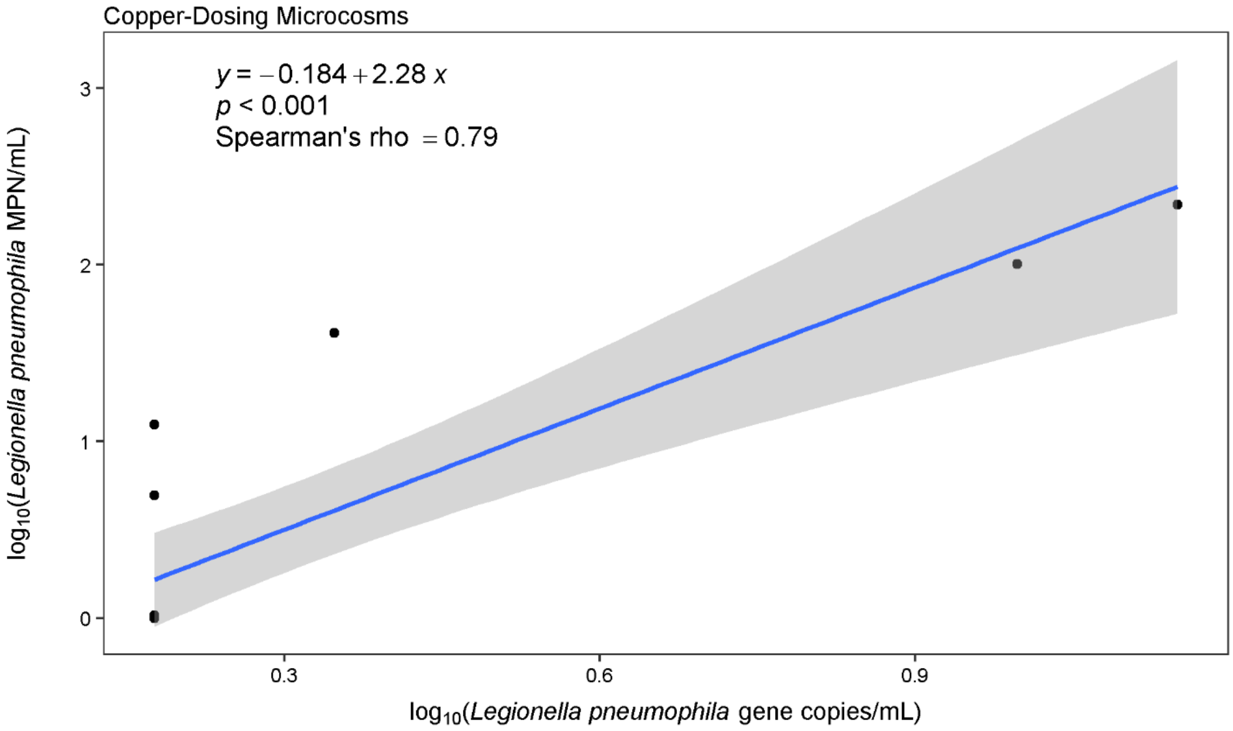

Supporting Information Figure 7. Log transformed bulk water culturable *Lp* (MPN/mL) determined by Legiolert™ vs *Neochlamydia* relative abundance (proportion of total reads per sample) determined by 16S rRNA gene amplicon sequencing for drinking water microcosms testing copper doses of 0-2000 µg/L Cu on Quincy *Lp* strain. Before log transforming, nondetects with ddPCR were set to the estimated limit of detection (1.5 gc/mL). Additionally, nondetects using LegiolertTM, values were set to the limit of detection (1 MPN/mL) for the non-potable 1 mL protocol. ddPCR values represent the average concentration amongst technical triplicates Both datasets [ddPCR (gc/mL) and Legiolert™ (MPN/mL)] include 5 copper levels × 3 microcosms × 3 sampling events = 45 measurements, which were averaged across events to yield 15 data points (one per microcosm).

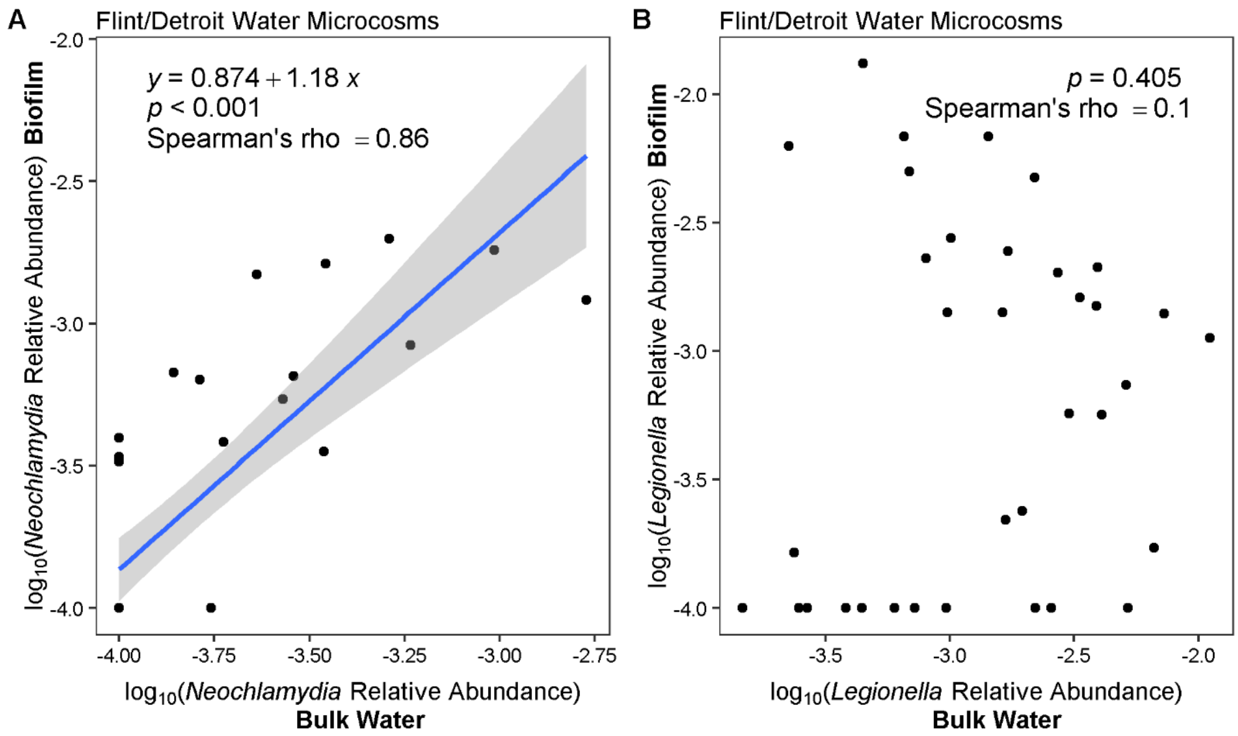

Supporting Information Figure 8. Log transformed *Neochlamydia* (A) and *Legionella* (B) relative abundance in bulk water versus biofilm for Flint/Detroit study (n=35). Where 0.0001 is added to each relative abundance to avoid log transforming 0. Biofilm and bulk water data were derived from 35 microcosms × 1 event (one per microcosm) = 35 data points.

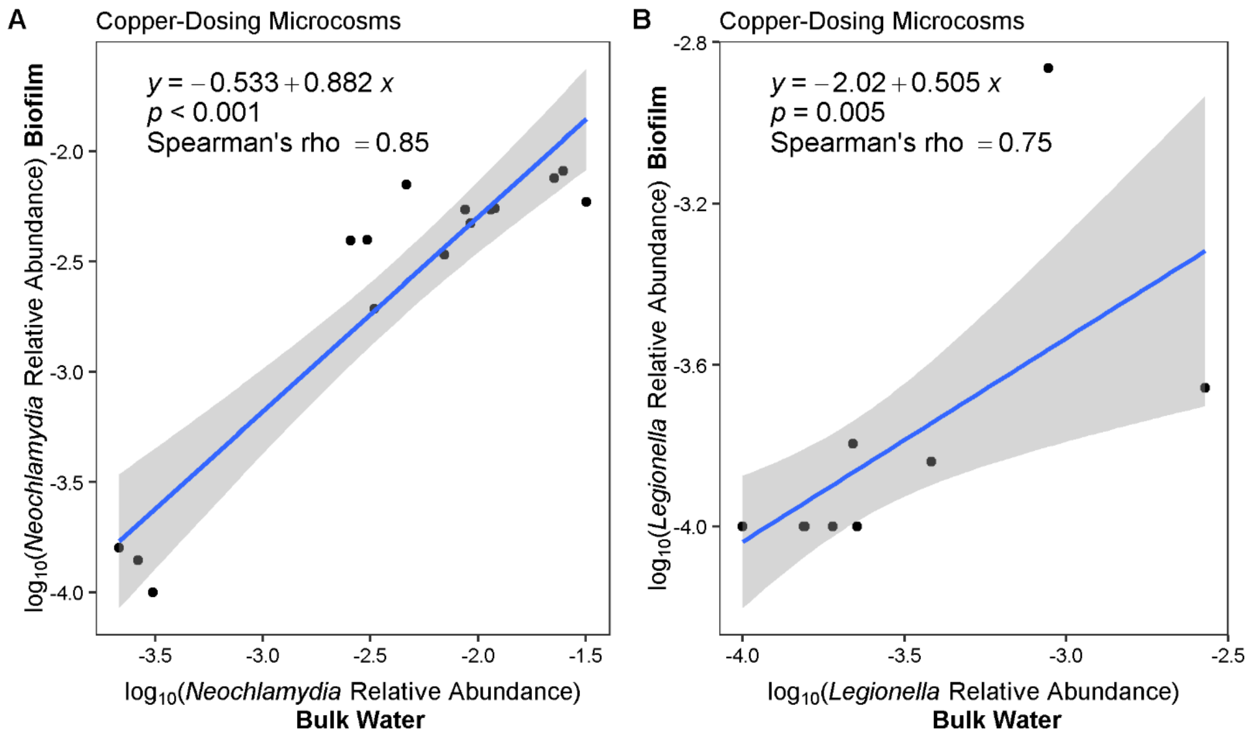

Supporting Information Figure 9. Log transformed *Neochlamydia (A)* and *Legionella (B) r*elative abundance in bulk water versus biofilm for Copper-Dosing study (n=15). Where 0.0001 is added to each relative abundance to avoid log transforming 0. For bulk water, data were derived from 5 copper levels × 3 microcosms × 3 sampling events = 45 samples, which were averaged by event to yield 15 data points (one per microcosm). For biofilm, 5 copper levels × 3 microcosms × 2 swabs = 30 swab samples, which were averaged to 15 data points (one per microcosm).

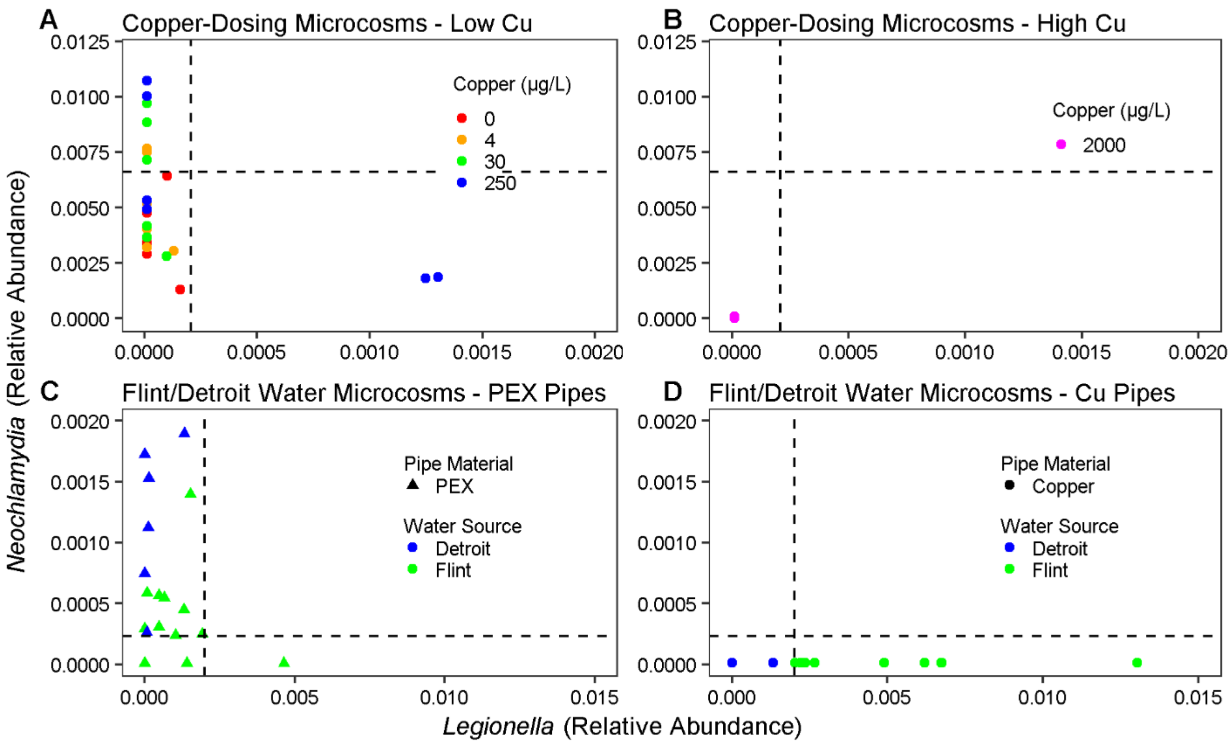

Supporting Information Figure 10: Biofilm *Legionella* vs. *Neochlamydia* relative abundance (proportion of total reads per sample) estimated by 16S rRNA gene amplicon sequencing of drinking water microcosms. (A) Copper-Dosing microcosms inoculated with the Quincy *Lp* strain and treated with 0-250 µg/L Cu [4 copper levels × 3 microcosms × 2 swabs = 24 data points] or (B) 2000 µg/L Cu [1 copper level × 3 microcosms × 2 swabs = 6 data points]. Flint/Detroit microcosms containing Flint or Detroit water sources (indicated in legend) and (C) PEX pipes [Flint (12 microcosms × 1 swab = 12 data points) and Detroit (6 microcosms × 1 swab = 6 data points)] or (D) copper pipes [Flint (11 microcosms × 1 swab = 11 data points) and Detroit (6 microcosms × 1 swab = 6 data points)]. Thresholds for "high" and "low" were determined using the average *Neochlamydia* and *Legionella* concentrations in the bulk water and biofilm across the Copper-Dosing microcosms (A and B), and the Flint/Detroit microcosms (C and D).

REFERENCES

1. International Standard Organization. Water Quality ─ Detection and Enumeration of Legionella. ISO 11731; International Standards Organization: Geneva, **1998**.

2. Martin, R. L.; Strom, O. R.; Song, Y.; Didier Mena-Aguilar; Rhoads, W. J.; Pruden, A.; Edwards, M. Copper Pipe, Lack of Corrosion Control, and Very Low PH May Have Influenced the Trajectory of the Flint Legionnaires' Disease Outbreak. ACS ES&T Water **2022**, 2 (8), 1440–1450. DOI: 10.1021/acsestwater.2c00182.

3. Smeltz, R. E.; Roman, F. A., Jr.; Byrne, T.; Finkelstein, R.; Song, Y.; Pruden, A.; Edwards, M. A. Influence of Copper Dose on Mycobacterium avium and Legionella pneumophila Growth in Premise Plumbing. bioRxiv **2025**, DOI: 10.1101/2025.03.20.644365.

4. Mena-Aguilar, D. P. Proteomic and Genomic Characterization of the Influence of Copper on Legionella pneumophila and the Drinking Water Microbiome; Ph.D. Dissertation, Virginia Tech, Blacksburg, VA, **2022**; Chapter 4. https://vtechworks.lib.vt.edu/items/1a9f981d-dfc9-4eb5-a43d-43ebbe9a1b7c (accessed 2025-03-04).

5. Bolyen, E.; Rideout, J. R.; Dillon, M. R.; et al. Reproducible, Interactive, Scalable and Extensible Microbiome Data Science Using QIIME 2. Nat. Biotechnol. **2019**, 37 (8), 852–857. DOI: 10.1038/s41587-019-0209-9.

6. Callahan, B. J.; McMurdie, P. J.; Rosen, M. J.; Han, A. W.; Johnson, A. J. A.; Holmes, S. P. DADA2: High-Resolution Sample Inference from Illumina Amplicon Data. Nature Methods **2016**, 13 (7), 581–583. DOI: 10.1038/nmeth.3869.

7. Bokulich, N. A.; Kaehler, B. D.; Rideout, J. R.; Dillon, M.; Bolyen, E.; Knight, R.; Huttley, G. A.; Gregory Caporaso, J. Optimizing Taxonomic Classification of Marker-Gene Amplicon Sequences with QIIME 2'S Q2-Feature-Classifier Plugin. Microbiome **2018**, 6 (1). DOI:10.1186/s40168-018-0470-z.

8. Quast, C.; Pruesse, E.; Yilmaz, P.; Gerken, J.; Schweer, T.; Yarza, P.; Peplies, J.; Glöckner, F. O. The SILVA Ribosomal RNA Gene Database Project: Improved Data Processing and Web-Based Tools. Nucleic Acids Research **2012**, 41 (D1), D590–D596. DOI: 10.1093/nar/gks1219.

9. R Core Team. R: A Language and Environment for Statistical Computing; R Foundation for Statistical Computing: Vienna, Austria, **2021**. https://www.R-project.org/ (accessed 2025-03-10).

10. Posit Team. RStudio: Integrated Development Environment for R; Posit Software, PBC: Boston, MA, **2024**. https://www.posit.co/ (accessed 2025-03-10).

11. Weiss, S.; Xu, Z. Z.; Peddada, S.; Amir, A.; Bittinger, K.; Gonzalez, A.; Lozupone, C.; Zaneveld, J. R.; Vázquez-Baeza, Y.; Birmingham, A.; Hyde, E. R.; Knight, R.

Normalization and Microbial Differential Abundance Strategies Depend upon Data Characteristics. *Microbiome* **2017**, *5* (1). DOI: 10.1186/s40168-017-0237-y.

12. McMurdie, P. J.; Holmes, S. Waste Not, Want Not: Why Rarefying Microbiome Data Is Inadmissible. PLoS Computational Biology **2014**, 10 (4). DOI:10.1371/journal.pcbi.1003531.

13. Love, M. I.; Huber, W.; Anders, S. Moderated Estimation of Fold Change and Dispersion for RNA-Seq Data with DESeq2. Genome Biology **2014**, 15 (12). DOI:10.1186/s13059-014-0550-8.

14. Oksanen, J.; Guillaume Blanchet, F.; Friendly, M.; Kindt, R.; Legendre, P.; McGlinn, D.; Minchin, P. R.; O'Hara, R. B.; Simpson, G. L.; Solymos, P.; Stevens, M. H. H.; Szoecs, E.; Wagner, H. vegan: Community Ecology Package; R package version 2.6-4, **2022**. https://CRAN.R-project.org/package=vegan (accessed 2025-03-01).

15. Nazarian, E. J.; Bopp, D. J.; Saylors, A.; Limberger, R. J.; Musser, K. A. Design and Implementation of a Protocol for the Detection of Legionella in Clinical and Environmental Samples. Diagnostic Microbiology and Infectious Disease **2008**, 62 (2), 125–132. DOI: 10.1016/j.diagmicrobio.2008.05.004.

16. Vouga, M.; Diabi, H.; Boulos, A.; Baud, D.; Raoult, D.; Greub, G. Antibiotic Susceptibility of Neochlamydia Hartmanellae and Parachlamydia Acanthamoebae in Amoebae. *Microbes and Infection* **2015**, *17* (11–12), 761–765. DOI:10.1016/j.micinf.2015.08.002

17. Rački, N.; Morisset, D.; Gutierrez-Aguirre, I.; Ravnikar, M. One-step RT-droplet digital PCR: a breakthrough in the quantification of waterborne RNA viruses. *Analytical and Bioanalytical Chemistry* **2014**, *406* (3), 661-667. DOI: 10.1007/s00216-013-7476-y.

18. Venables, W. N.; Ripley, B. D. Modern Applied Statistics with S, 4th ed.; Springer: New York, 2002; ISBN 0-387-95457-0.

19. Wang, H.; Edwards, M.; Falkinham, J. O.; Pruden, A. Molecular Survey of the Occurrence of Legionella Spp., Mycobacterium Spp., Pseudomonas Aeruginosa, and Amoeba Hosts in Two Chloraminated Drinking Water Distribution Systems. Applied and Environmental Microbiology **2012**, 78 (17). DOI:10.1128/aem.01492-12.

20. NCBI GenBank. Neochlamydia sp. CRIB37 16S ribosomal RNA gene, partial sequence (EU683885.1). https://www.ncbi.nlm.nih.gov/nuccore/EU683885.1 (accessed 2025-03-02).
